# The impact of contraceptives on the vaginal microbiome in the non-pregnant state

**DOI:** 10.3389/frmbi.2022.1055472

**Published:** 2023-01-30

**Authors:** Cassandra Bakus, Kelly L. Budge, Nicole Feigenblum, Melissa Figueroa, Antonia P. Francis

**Affiliations:** ^1^ Hackensack Meridian School of Medicine, Hackensack Meridian Health Network, Nutley, NJ, United States; ^2^ Department of Obstetrics and Gynecology, Hackensack Meridian Health Network, Hackensack University Medical Center, Hackensack, NJ, United States

**Keywords:** contraception, lactobacilli, vaginal microbiome, dysbiosis, hormones

## Abstract

The vaginal microbiome exists in a dynamic state and its disruption, by diminution of *Lactobacillus* concentrations, can induce a state of microbial imbalance with significant health consequences, such as increased risk of sexually transmitted infection (STI) acquisition, preterm labor, and low birth weight babies. This delicate balance of microbes can be affected by many processes such as mechanical practices (i.e. douching) and hormonal changes: physiologic (i.e. menstrual cycle, menopause, puberty), pathologic (i.e. PCOS), and exogenous (i.e. contraceptives). Contraceptives fall into mechanical and hormonal categories, both of which prevent unintended pregnancy. The mechanical contraceptives of spermicides, diaphragms, and cervical caps alter the vaginal ecosystem, with spermicides being linked to an increased risk of vaginal dysbiosis. The impacts of Copper T intrauterine devices (Cu-IUDs) and hormonal contraceptives on the vaginal microbiome are contradictory. A better understanding and consensus of how contraceptive methods affect the vaginal microbiome is needed.

## 1 Introduction

A complex relationship between microbes and their hosts has been well documented over the last two decades shaped by the advent of new technologies. High throughput sequencing has allowed scientists to identify microbial communities while studying their dynamic interactions through genomic, proteomic, transcriptomic, and metabolomic to further understand their intricate relationships (Pavlova et al., 2002; Antonio et al., 1999) Within the human reproductive system, there is a mutualistic relationship between host and microbes known as the *vaginal microbiome*. Disruption of this balance is known as dysbiosis. The vaginal microbiome considers the microbial composition of the host’s vagina, whose composition has historically been thought to be dominated by the *Lactobacillus* genus of gram-positive anaerobes in the state of normalcy (Burton and Reid, 2022). Recent advances in genome sequencing and metagenomic technologies have enabled scientists to observe regional and racial variations in vaginal microflora, questioning our concept of normalcy (Drell et al., 2013; Martínez-Peña et al., 2013; Pendharkar et al., 2013). While it is important to acknowledge this emerging information and the subjectivity of historical normalcy, the data remains unclear and does not provide strong evidence to redefine normalcy of the vaginal microbiome.

The vaginal microbiome exists as a dynamic environment. A multitude of factors influences this, including pregnancy (see 2.2), age (see 2.3), douching (see 2.5), menstruation (see 2.6), and contraception (see 3). Approximately 65% of women in the United States of America between the ages of 15-49 use a form of contraception at a given moment in time, and nearly all sexually active U.S. women have used a form of contraception at some point during their reproductive years (Center for Disease Control and Prevention, 2018; Kaiser Family Foundation, 2021). Given the high prevalence of contraceptives, it is necessary to understand the influence of contraceptives on alterations in vaginal microbiome and it is important to improve research on this topic to provide appropriate counseling for patients.

## 2 Alterations in the vaginal microbiome

### 2.1 Defining dysbiosis

Vaginal dysbiosis is broadly defined by a decrease in *Lactobacillus* species population in the vagina. This causes an increase in pH, which is associated with negative health effects, such as bacterial vaginosis (BV), which has been linked to preterm labor and increased risk of acquiring sexually transmitted infections and vulvovaginal infections (Hillier et al., 1995; Brotman et al., 2010; Kalia et al., 2020; Ravel et al., 2021). The exact mechanism that causes vaginal dysbiosis is still unknown (van de Wijgert et al., 2014). To better understand dysbiotic states, it is imperative to know the composition of a healthy vaginal microbiome.

The vaginal microbiome is dominated by *Lactobacillus*, which produces lactic acid to create an acidic environment in the vagina (O'Hanlon et al., 2013; Tachedjian et al., 2017). This confers an innate immunity within the vagina to fight off pathogenic bacteria and to further allow *Lactobacillus* to proliferate. Such mechanisms include acidifying the bacterial cytoplasm leading to cell death and acting as a permeabilizer of gram-negative bacterial cell membranes (Alakomi et al., 2000).

### 2.2 Early impact of the vaginal microbiome

The exact mechanism by which the vaginal microbiome is initially formed is unknown. It is hypothesized that individuals can be predisposed to dysbiosis through various means. One theory states that vaginal imbalance can be impacted with*in utero* life. Findings from Collado et al. suggest that the fetal-maternal interface can be an important source of seeding for the gut microbiome, but its effects on the fetal vaginal microbiome have yet to be investigated (Collado et al., 2016).

Additionally, different birthing methods have been shown to impact the composition of the intestinal microbiome of the neonate. The intestinal microbiome of infants is dominated by maternal fecal bacteria, but neonates born *via* cesarean lack this exposure (Carlsson and Gothefors, 1975; Korpela et al., 2020; Song et al., 2021). The short- and long-term health consequences of this intervention are not fully understood.

### 2.3 Changes of the vaginal microbiome with time

A time frame that is marked by distinct changes in the vaginal microbiome is between pre-and post-puberty. Pre-pubescent individuals have a greater diversity of anaerobes, aerobes, and enteric bacteria. The onset of menarche is noted by *Lactobacillus* predominance, which is seen in most reproductive-aged women. This is likely due to the increase of estrogen which produces more intracellular glycogen supporting *Lactobacillus* growth (Porter et al., 2016). As the organ continues to develop, one of the ways in which the vagina maintains homeostasis is vaginal discharge. Vaginal discharge is a collection of mucus from the cervix, which is protective as it removes potentially pathogenic material as it travels down the vaginal canal. However, the self-cleaning properties are not a perfect defense and pathogenic bacteria can colonize and overgrow in the vagina.

### 2.4 Bacterial vaginosis

BV is a state of dysbiosis characterized by foul-smelling vaginal odor and thin, gray-white vaginal discharge (Tachedjian et al., 2017). Although the inciting entity for BV remains controversial, it typically presents with a decreased population of *Lactobacillus* and high concentrations of anaerobic bacteria including *Gardnerella vaginalis*, *Prevotella* spp., *Atopobium vaginae*, *Sneathia* spp., and other BV-associated bacteria (BVAB) (Muzny et al., 2020). BV is the most common vaginal imbalance (Ahmadnia et al., 2016) affecting 21.2 million (29.2%) women in the United States aged 14-49 (Koumans et al., 2007). Adverse health outcomes of BV are associated with pre-term delivery of a low-birth-weight baby (Hillier et al., 1995), increased risk of acquiring sexually transmitted infections such as trichomonal, gonococcal, and chlamydial infections (O'Hanlon et al., 2013), and a controversial effect on fertility (Liversedge et al., 1999; Campisciano et al., 2017).

### 2.5 Douching

Although BV is one of the most common causes of dysbiosis, there are other mechanisms that can cause the imbalance. A mechanical mechanism that can cause dysbiosis in reproductive-age women is douching. Douching has been shown to decrease hydrogen peroxide producing *Lactobacillus*, therefore, increasing the vaginal pH (Ness et al., 2002; Beigi et al., 2005). Women with biologic vaginas and who douche regularly have a higher risk of developing BV (Ness et al., 2002).

### 2.6 Hormones

Hormones can affect the bacterial composition of the vaginal microbiome. An example of a high hormonal state is Polycystic Ovary Syndrome (PCOS), with increased levels of testosterone and alterations in estrogen and progesterone levels. Women with PCOS were found to be colonized with a higher abundance of *Mycoplasma* and *Prevotella* with a lower prevalence of *Lactobacillus crispatus* (Hong et al., 2020).

A physiologic state that is characterized by hormonal fluctuations is the menstrual cycle. The two organs involved in the menstrual cycle are the uterus and ovaries. The uterus has three phases: menstrual, proliferative, and secretory while the ovaries have two: follicular and luteal. These cyclical phases of the two organs occur simultaneously, with the proliferative and follicular phases being dominated by estrogen and the secretory and luteal by progesterone. It should be noted that the menstrual cycle is usually 28 days but can vary among individuals (Baker and Driver, 2007).

Hormonal influences from the menstrual cycle have been documented to influence the vaginal microbiome as well. There is not a consensus on exactly which bacteria are affected during specific phases in the menstrual cycle. Some women’s microbiome remains stable throughout the menstrual cycle, and others experience a temporal shift in the dominance of bacterial populations during menses, which returns to normal once menses stop (Song et al., 2020; Critchley et al., 2020). Other studies report linear changes in bacterial populations throughout the menstrual cycle (Eschenbach et al., 2000).

*Lactobacillus* is a key player in the vaginal microbiome. Its predominance provides protection against an array of pathogens, and its disruption is associated with several diseased states. Discerning which practices are harmful or hurtful to its colonization provides insights to improve vaginal health. With the popularity of contraceptive use, it is important to understand how this practice can be impacted.

## 3 What we know about contraception and the vaginal ecosystem

There are many methods of contraception that are often focused on preventing unintended pregnancies. Although a non-hormonal contraceptive pill (YCT529) for men has recently started clinical trials, currently available male contraception is conventionally focused on condoms and vasectomy. Options for persons with female reproductive organs are more comprehensive and include reversible, hormonal, mechanical, and permanent contraception. Choosing a method of contraception is often a personal decision including the individual’s subjective characteristics and the availability and usage of the method. While each method brings its own advantages and risks, there is a lack of discussion and understanding of the effects of contraceptives on the vaginal microbiome (**Table 1**).

**Table 1:** Summary of the different forms of contraception and their known effects on the vaginal microbiome.

Form of Contraception	Effect on Vaginal Microbiome
Non-Hormonal	
Spermicides	Decreases the prevalence of *Lactobacillus* species likely secondary to the detergent-like action on the vaginal epithelium and flora (Wilkinson et al., 2002; Schreiber et al., 2006).
Mechanical contraceptives	
Condoms	*In adolescence*: conversion to an inflammatory state but has minimal effect on the vaginal flora (Farr Zuend et al., 2021). *In adults*: vaginal dermatitis and vulvovaginitis (Fosch et al., 2018).
Diaphragm and cervical caps	Provokes mechanical and chemical disturbances. *Enterococcus* and *E. coli* are significantly increased one week after use of either (Gupta et al., 2000).
Sponge	No data
Non-hormonal IUD	Contradicting data regarding whether there is a change to the vaginal flora: •No association between the Cu-IUD and BV (Landolt et al., 2018) •Higher BV prevalence in those with Cu-IUD ▪Secondary to the presence of the foreign object which promotes colonization (Srinivasan et al., 2010; Achilles et al., 2018; Peebles et al., 2021) ▪Increased volume and duration of menstruation leads to increased availability of iron-containing metalloproteins which allows for the growth of *G. Vaginalis* to persist (Srinivasan et al., 2010)
**Hormonal**	
Levonorgestrel-IUD	Promotes a stable microbiome through the steady release of progestin (Jacobson et al., 2014; Hashway et al., 2014; Bassis et al., 2017; Achilles et al., 2018; Pelzer et al., 2018).
Etonogestrel implant	Has not been found to cause significant change (Achilles et al., 2018).
Depot medroxyprogesterone	Promotes a stable microbiome (van de Wijgert et al., 2013; Mitchell et al., 2014; Brooks et al., 2017).
Combined oral contraceptives	Promotes a stable microbiome (Aagaard et al., 2012; van de Wijgert et al., 2013; Brooks et al., 2017).
Progestin-only contraceptives	Minimal data with some demonstrating an increase in aerobic vaginitis and vaginal atrophy (Kazi et al., 2012; Balle et al., 2020; Bastianelli et al., 2021).
NuvaRing	Promotes *Lactobacilli* predominance and decreases *G. Vaginalis* in a high bacterial vaginosis population (Jacobson et al., 2014).
Contraceptive Patch	No data

BV, bacterial vaginosis; Cu-IUD, copper IUD.

### 3.1 Spermicides and mechanical contraceptive effect on the vaginal microbiome

Mechanical contraception is often referred to as the barrier method. The diaphragm, cervical cap, male and female condoms, and spermicide form a physical block, preventing the fertilization of eggs by sperm. Often overlooked and under-emphasized is their influence on the vaginal microbiome.

#### 3.1.1 Spermicides

Spermicides are sold as creams, films, foams, gels, or suppositories that are designed to kill sperm and/or block entry of sperm into the cervix. However, the product has been linked to vaginal microbiome alterations. The most common form of spermicide, Nonoxynol-9, significantly alters the vaginal microbiome leading to an apparent lack in *Lactobacillus* (Schreiber et al., 2006). This is a dose and exposure-dependent change, likely due to the nonspecific, detergent-like action on the vaginal epithelium and flora, enhancing the susceptibility of the space to vaginal irritation and allergic vaginitis. Additionally, the endothelial disruption may provide a viral entryway, as there is evidence of harm through genital lesions with Nonoxynol-9 use (Wilkinson et al., 2002). Despite the increased risk of dysbiosis and clinical injury, Nonoxynol-9 has continued to be sold as an over-the-counter (OTC) drug in creams, gels, foams, and condom lubricants for more than 30 years.

While the surfactant Nonoxynol-9 disrupts cell membranes, other spermicides impede sperm function in a less extreme fashion, though similar issues are reported. Cellulose sulfate impedes sperm penetration into cervical mucus but increases mucosal inflammation (Pellett Madan et al., 2015). Similar outcomes of the cellulose sulfate gel on an increased risk of HIV acquisition have also been reported when compared to the use of Nonoxynol-9 (Van Damme et al., 2008). Phexxi, a more recent addition to the spermicide market, is believed to have a gentler effect on the vaginal epithelium with its acid-based formulation. This acidic composition disrupts sperm and may also disrupt the vaginal microbial composition, reflecting the common side effects of yeast and urinary tract infections (Svoboda, 2020).

#### 3.1.2 Mechanical contraceptives

Male and female condoms are sheath barrier devices often made of latex that may be used in conjunction with lubricants or spermicides. The use of condoms has been associated with a conversion of the vaginal microbiome to an inflammatory state. In adolescent girls, condom use has been associated with changes in functional metabolic pathways in vaginal bacteria and inflammatory processes, but without significant changes in the overall vaginal microbiome (Farr Zuend et al., 2021). These functions are related to protein translation and fructose and mannose metabolism, which are important pathways for energy production by *Lactobacillus*. In adults, condom use is correlated with the presence of vaginal dermatitis, allergic and irritant vulvovaginitis, and inflammation, likely due to the influence of either the latex or the spermicide as some condoms come coated with spermicide (Fosch et al., 2018). The vulvar mucosa is susceptible to irritants and mechanical disturbances due to its composition of hormone-responsive nonkeratinized epithelium. Condom use may function as both a mechanical and chemical disturbance promoting vaginitis, an inflammatory state, which can further lead to microbiome disturbances.

Diaphragms and Cervical caps function as physical barriers by covering the cervix. Both contraceptive methods require the co-use of spermicides, and both have been shown to clearly alter the normal vaginal ecosystem. *Enterococcus* and *E. coli* species have been found to be significantly increased one week after initiation of either cervical caps or diaphragm use (Gupta et al., 2000). These methods may provoke both mechanical and chemical disturbances to the vaginal microbiome, but the exact mechanism is unknown.

Contraceptive sponges similarly cover the cervix and contain Nonoxynol-9 spermicide to prevent pregnancy. However, there is no available data on the effect of the sponge on the vaginal microbiome in humans although one brand remains available on the US market.

Mechanical contraception can cause both physical and irritant injury to the vaginal epithelium and mucosa which can consequently increase susceptibility to further damage.

### 3.2 Nonhormonal intrauterine device (IUD)

Copper T intrauterine device (Cu-IUD) is a nonhormonal, metal device that is placed in the uterus and can reside there for up to 10 years as an effective form of birth control. Contradicting evidence has been reported regarding their association with vaginal dysbiosis. While Kancheva Landolt et al. (2018) reported there was no association between Cu-IUD use and BV, Peebles et al. (2021) reported a 1.28-fold increase in BV compared to those without or with another nonhormonal contraception. It should be noted that the study by Landolt et al. (2018) focused on a population in Thailand with a high BV prevalence among all their groups with or without contraception initiation. Another study executed in Zimbabwe also found a positive association between BV and Cu-IUD use, notable by increased colonization with the BV-associated microbiota *G. vaginalis* and *A. vaginae* (Achilles et al., 2018).

**Figure 1:**
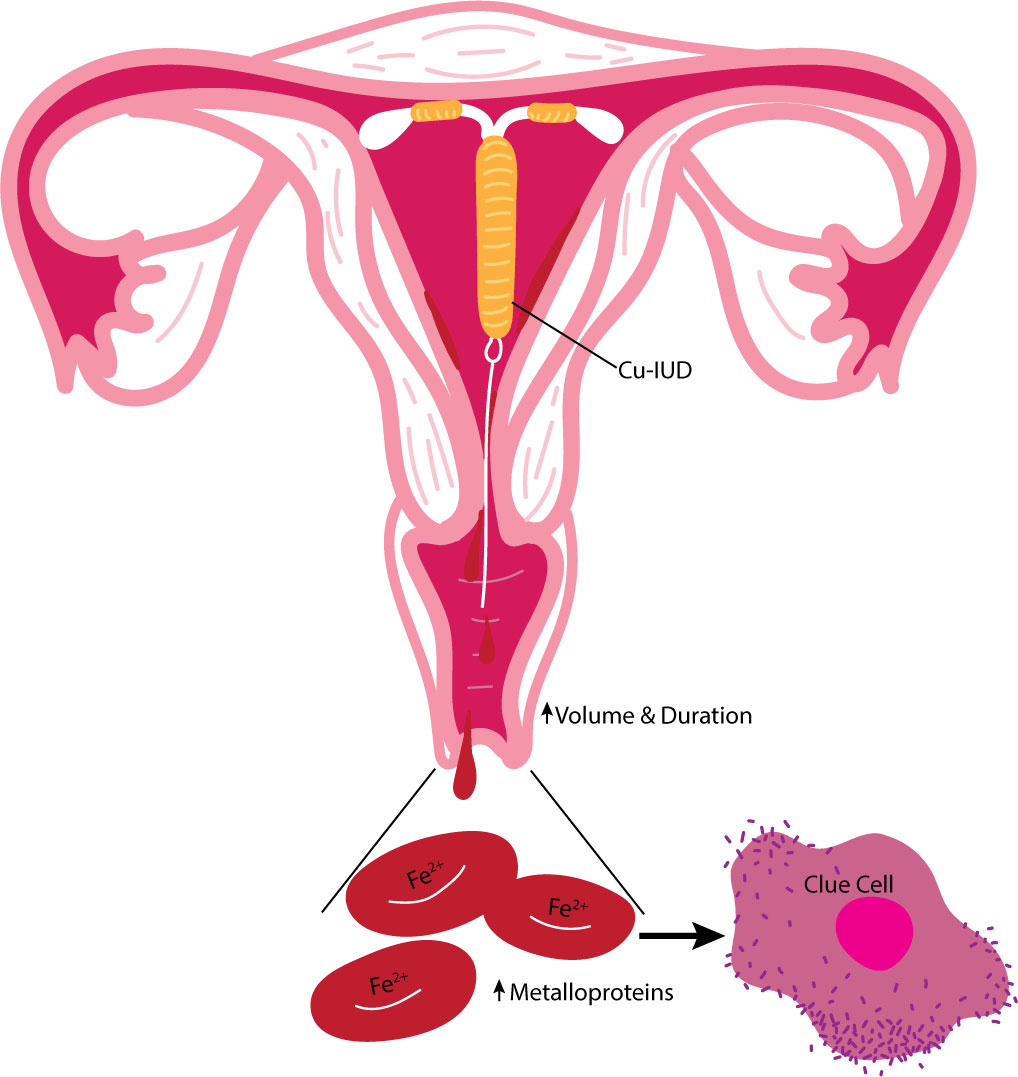
Summary of the proposed influence of the Cu-lUD on the vaginal environment. Cu-IUDs are associated with heavier menstrual cycles likely caused by copper's influence of vascular changes increasing the volume and duration of bleeding. The increased availability of iron-containing metalloproteins in erythrocytes due to heavy menstruation promotes colonization of BV associated microbiota such as *G. vaginalis.*.

There are two predominating theories as to how Cu-IUDs impact the vaginal microbiome. The continued presence of the foreign object may promote colonization of BV-associated microbiota. However, the disruption to the vaginal microbiome that is seen with Copper IUDs is not similarly seen in hormonal IUD methods discussed in detail below, suggesting that this reaction is specific to the copper release. Alternatively, the influence of Cu-IUD initiation on the individual menstruation cycle may contribute to vaginal dysbiosis. A decrease in the predominant *Lactobacillus* species and increase in *G. vaginalis* often occurs during menstruation denoting a temporal variability in the microbiota of the human vagina (Srinivasan et al., 2010). As increased volume and duration of menstruation often accompanies Cu-IUD initiation, the growth of *G. vaginalis* may be allowed to persist to the point of dysbiosis due to the increased availability of iron-containing metalloprotein in erythrocytes (**Figure 1**).

### 3.3 Effects of hormones on vaginal microbiome

#### 3.3.1 The hormonal state and its implications with the vaginal microbiome

Hormonal contraceptives are a common form of birth control composed of a synthetic form of progestin with or without estrogen, which inhibits ovulation through suppression of the hypothalamic-pituitary-gonadal (HPG) axis. Although the delivery system may vary, different forms have similar effects, often accompanied by side effects indicating the hormones’ systemic capabilities.

To understand estrogen and progesterone’s impact on the vaginal microbiome, it is beneficial to understand changes to the system during a condition of decreased estrogen and progesterone production like menopause. During menopause, low hormone levels result in a decreased glycogen deposition on the vaginal epithelium, leading to less free glycogen availability for *Lactobacilli* nutrition. In comparison to a *Lactobacillus*-predominant vaginal microbiome in premenopausal American women (83%), post-menopausal women experience a statistically significant difference in the composition of the taxonomic distribution, decreasing to 54% (Brotman et al., 2014). A similar decrease of 63.2% to 23.7% was observed in Korean women when comparing pre- and post-menopausal taxonomic distributions, respectively (Kim et al., 2021). Post-menopausal vaginal microbiomes are additionally associated with increased diversity in microbe species and increased pH. Lactic acid from metabolized glycogen during reproductive age maintains an acidic vaginal pH of 3.8-4.2, suppressing the overgrowth of infectious organisms. This contrasts with postmenopausal women taking hormone replacement therapy (increased circulating estrogen) who are more likely to have majority vaginal *Lactobacilli* like those of reproductive age (Dahn et al., 2008).

Juxtaposing the low hormonal state of menopause, pregnancy posits a condition of high progesterone for further characterization of the hormonal influences on the vaginal microbiome. The vaginal microbiome signature in pregnancy is distinct from non-pregnant. Overall diversity and richness are reduced in pregnancy, with a dominance of *Lactobacillus* species *iners*, *crispatus*, *jensenii*, and *johnsonii* (Aagaard et al., 2012). *Lactobacilli* not only produce lactic acid, but they may also produce hydrogen peroxide (H_2_O_2_). which is bactericidal. *L crispatus* is a strong H_2_O_2_ producer. This activity of acidity and H_2_O_2_ production reflects an innate immune defense against pathogenic organisms and may be critical during pregnancy to prevent dysbiotic vaginal colonization and ascending infection.

#### 3.3.2 Exogenous hormones

Exogenous hormonal administration similarly saw an emergence of *L. crispatus* as the dominant phylotype in both the endometrial and the endocervical samples (Pelzer et al., 2018). In Pelzer et al, patients utilized a levonorgestrel-releasing IUD (hormonal IUD) for consistent release of progestin, and other studies have similarly corroborated that consistent hormonal dosing promotes a stable vaginal microbiome (Jacobson et al., 2014; Hashway et al., 2014; Achilles et al., 2018; Pelzer et al., 2018).

The use of combined oral contraceptives (COCs) (Aagaard et al., 2012) and depot-medroxyprogesterone acetate (DMPA, i.e. ‘the shot’) (Mitchell et al., 2014) have revealed similar results of a stable vaginal microbiome dominated by H_2_O_2_-producing bacteria of the *Lactobacillus* genus. COCs and DMPA impart exogenous hormones within the system and are associated with overall decreased BV-associated vaginal dysbiosis (van de Wijgert et al., 2013; Brooks et al., 2017). COC Etonogestrel implanted contraception has not been found to cause significant change (Achilles et al., 2018). There is minimal data on the effect of progesterone-only-pills (POP or ‘mini-pill’) on the vaginal microbiome, but some research has demonstrated no change in the BV rates, but increased rates of aerobic vaginitis and vaginal atrophy (Kazi et al., 2012; Balle et al., 2020). This is thought to be due to the increased bleeding that can occur with POPs compared to COC and hormonal IUDs (Bastianelli et al., 2021). The NuvaRing, an estrogen-containing vaginal ring, has been demonstrated to promote *Lactobacilli* predominance and decreasing *G. vaginalis* in a high-BV population (Crucitti et al., 2018). No data on the effect of the contraceptive patch on vaginal flora has been documented.

However, it is important to note that contradicting evidence has been reported regarding hormonal contraceptive use. Donders et al. reported COC and Hormonal IUD users had the same bacterial composition as non-contraceptive users (Donders et al., 2017). While Brooks et al. noted Hormonal IUD use was accompanied by several taxa typically associated with a dysbiotic vaginal microbiome including *Prevotella* (Brooks et al., 2017). The same study noted that women using DMPA and hormonal IUDs were no more or less likely to be colonized by H_2_O_2_-producing *Lactobacillus* than women using condoms (Brooks et al., 2017). Achilles et al. (2018) further found no significant changes in beneficial *Lactobacillus* species over 180 days after initiation of injectable (DMPA, norethisterone enanthate, or medroxyprogesterone acetate and Ethinyl estradiol) or implanted (levonorgestrel or etonogestrel) contraception. Additionally, a study conducted by Bassis et al. (2017) found that hormonal IUD had no effect on vaginal microbiome composition over a 12-month period.

**Figure 2:**
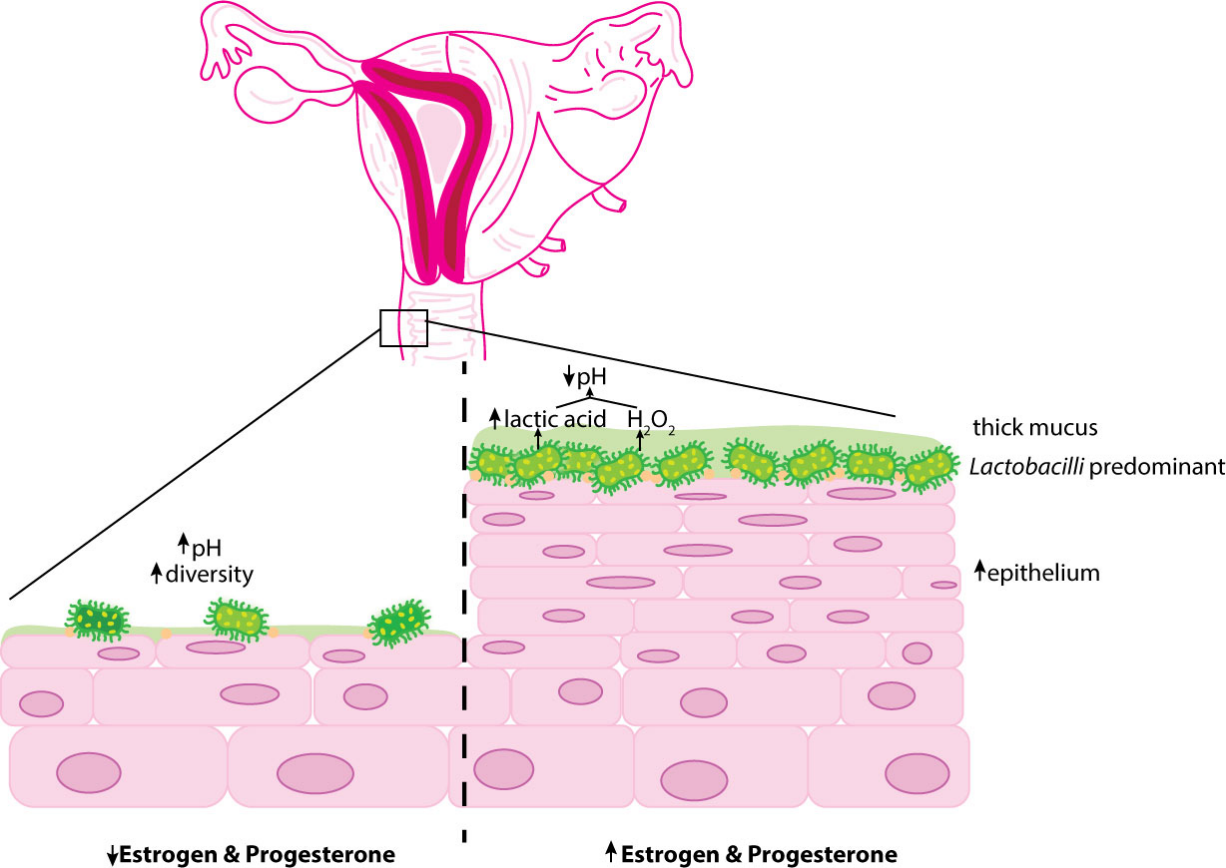
Overview ofthe influence of hormonal contraception on the vaginal epithelium. Estrogen and progesterone encourage thickening of the vaginal mucosa and glycogen deposition. Glycogen presence preferentially selects for glucose-fennenting microorganisms such as *Lactobacilli. Lactobacilli* metabolize glycogen into lactic acid and fonn H_2_O_2_ promoting an acidic environment. Decreased hormonal systems (prepubertal and postmenopausal) are associated with reduced glycogen deposition increasing the diversity of vaginal microbe species and vaginal pH.

Another concern noted with hormonal contraception is that COC use may increase vaginal candidiasis (van de Wijgert et al., 2013). Additionally, the age of the individual using COC influenced alterations in the vaginal microbiota, although *Lactobacilli* remained predominant in all age categories (Kazi et al., 2012). Despite the contrasting data, the majority of the evidence supports that hormonal contraception influences a vaginal microbiome of decreased diversity and pH predominated by H_2_O_2_-producing bacteria of the Lactobacillus genus (Brooks et al., 2017; Song et al., 2020; Balle et al., 2020) (**Figure 2**).

## 4 Conclusions and future directions

The vaginal microbiome is an ever-adapting system that is predominated by *Lactobacillus*, which confers innate immunity by creating an acidic environment. When there is an imbalance, or dysbiosis, within the system it can lead to bacterial, viral, and fungal infection, vaginal atrophy, and vaginitis. Not only do alterations in weight, hormones, and pregnancy cause dysbiosis but external sources like douching, vaginal intercourse, and contraception impact the vaginal flora as well. With the high rate of contraception use in our population, it is vital to have a strong understanding of how these different methods may impact the vaginal microbiome.

Contraceptive counseling should be a shared decision-making process between patients and physicians considering patient history, physical exam findings, and patient desires from birth control that can then be used to arrive at the method that is best for the patient. Physicians counseling patients with recurrent infections or sensitivities may advise against spermicides, condoms, diaphragms, and cervical caps and recommend hormonal methods that may promote a more stable vaginal microbiome. Still, several areas require further understanding and research, including the impact of copper IUD and a consensus on the influence of various hormonal contraceptives on the vaginal microbiome. Better insight is needed to further understand the risks and benefits of each contraceptive option in relation to vaginal health so we can empower our patients to make informed decisions.

## Author contributions

KB, CB, and NF wrote the manuscript. MF and AF supervised the manuscript. All authors contributed to the article and approved the submitted version.
